# PD‐L1 Expression and Histopathological Features in EGFR‐Mutated Non‐Small Cell Lung Cancer: Implications for Immune Checkpoint Inhibitors After EGFR‐Tyrosine Kinase Inhibitors Resistance

**DOI:** 10.1111/1759-7714.70252

**Published:** 2026-02-09

**Authors:** Toshiyuki Sumi, Taiki Ishigooka, Keigo Matsuura, Takumi Ikeda, Yuichi Yamada, Kotomi Arioka, Hirofumi Chiba

**Affiliations:** ^1^ Department of Pulmonary Medicine Hakodate Goryoukaku Hospital Hakodate Hokkaido Japan; ^2^ Department of Respiratory Medicine and Allergology Sapporo Medical University School of Medicine Sapporo Japan; ^3^ Department of Surgical Pathology Hakodate Goryoukaku Hospital Hakodate Hokkaido Japan

**Keywords:** epidermal growth factor receptor, immune checkpoint inhibitors, non‐small cell lung cancer, tyrosine kinase inhibitors

## Abstract

**Background:**

Epidermal growth factor receptor‐mutated (EGFRm) non‐small cell lung cancer (NSCLC) responds well to EGFR tyrosine kinase inhibitors (EGFR‐TKIs), yet optimal therapy after resistance remains uncertain. Although immune checkpoint inhibitors (ICIs) show limited overall efficacy, heterogeneity in response by programmed cell death ligand 1 (PD‐L1) expression exists.

**Methods:**

We retrospectively evaluated 90 patients with advanced/recurrent EGFRm NSCLC treated with first‐line EGFR‐TKIs (October 2018–October 2023). Clinicopathological and radiologic features were compared by PD‐L1 tumor proportion score (< 50% vs. ≥ 50%). The primary analysis evaluated overall survival from first progression (post‐progression OS/PPS) using a time‐varying Cox model with start–stop intervals, modeling ICI as a time‐dependent exposure ICI(*t*) and testing the ICI(*t*) × PD‐L1 interaction.

**Results:**

The PD‐L1 ≥ 50% group (*n* = 26) more often had solid histology (62% vs. 8%), solid nodules on computed tomography (96% vs. 52%), and larger tumors (median 42.0 vs. 27.5 mm). Among 68 patients evaluable from *t*_0_, the ICI(*t*) × PD‐L1 interaction was significant (Wald *p* = 0.015; likelihood‐ratio *p* = 0.036). Stratum‐specific adjusted ICI effects suggested a detrimental association in PD‐L1 < 50% (hazard ratio [HR]: 3.11, 95% confidence interval [CI]: 0.94–10.30) but favorable association in PD‐L1 ≥ 50% (HR: 0.457, 95% CI: 0.138–1.512). In exploratory analyses of 22 ICI‐treated patients, PD‐L1 ≥ 50% showed higher response rates (64% vs. 9%) and longer time to treatment failure (3.4 vs. 1.4 months).

**Conclusions:**

High PD‐L1 expression in EGFRm NSCLC is associated with more aggressive morphologic features and modifies the association between post‐progression ICI and survival. These findings support PD‐L1‐informed selection after EGFR‐TKI failure, while prospective confirmation is needed.

## Graphical abstract

This retrospective study elucidates the clinical significance of PD‐L1 expression in EGFR‐mutated non‐small cell lung cancer. High PD‐L1 expression (TPS ≥ 50%) was significantly associated with solid histological patterns and radiological solid nodules. Furthermore, a time‐varying survival analysis revealed that PD‐L1 status modifies the efficacy of immune checkpoint inhibitors (ICIs) administered after EGFR‐TKI resistance. The findings suggest that patients with high PD‐L1 expression may derive survival benefits from post‐progression ICI therapy compared to the PD‐L1‐low population.

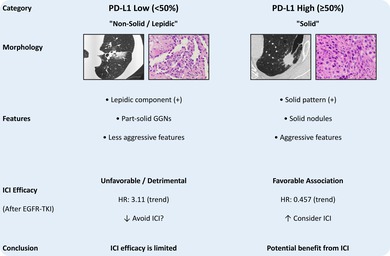

## 1 Introduction

Mutations in the *EGFR* gene are among the most well‐known driver mutations in non‐small cell lung cancer (NSCLC). EGFR tyrosine kinase inhibitors (EGFR‐TKIs) show high efficacy in EGFR‐mutated (EGFRm) NSCLC, with osimertinib widely used as first‐line therapy [1, 2]. However, beyond second‐line therapy, options are limited, with platinum‐based or single‐agent chemotherapy commonly employed.

Chemotherapy has remained the standard of treatment following EGFR‐TKI resistance, given the lack of demonstrated efficacy of immune checkpoint inhibitors (ICIs). A phase II trial of pembrolizumab in EGFRm, by programmed cell death ligand 1 (PD‐L1)‐positive NSCLC (PD‐L1 ≥ 1%) reported a 0% response rate in 10 patients, leading to early termination [3]. A pooled analysis of phase II/III trials comparing ICIs (nivolumab, pembrolizumab, atezolizumab) with docetaxel in second‐line NSCLC found no overall survival (OS) benefit in EGFRm cases (hazard ratio [HR]: 1.11, 95% confidence interval [CI]: 0.80–1.53, *p* = 0.54) [4].

Recently, new findings have emerged in PD‐L1‐positive, EGFRm NSCLC. One study reported an inverse correlation between PD‐L1 levels and EGFR‐TKI progression‐free survival (PFS) [5]. Another showed that ICI therapy was particularly effective in patients with high PD‐L1 expression and shorter EGFR‐TKI PFS [6]. The ATTLAS phase III trial comparing CBDCA+PTX + BEV + ATEZ versus platinum‐pemetrexed showed significant PFS improvement in the atezolizumab arm (HR: 0.62, 95% CI: 0.45–0.86, *p* = 0.004), although OS was not prolonged (HR: 1.01, 95% CI: 0.69–1.46, *p* = 0.975) [7]. A subgroup analysis suggested a trend toward improved OS in patients with PD‐L1 ≥ 50%, although this change was not significant. These findings suggest some EGFRm NSCLC patients, particularly those with PD‐L1 ≥ 50%, may benefit from ICI therapy [8, 9]. Given that ICIs typically cause fewer adverse events than chemotherapy in driver‐negative NSCLC [10], identifying responders after EGFR‐TKI resistance is clinically important.

EGFRm NSCLC usually has low PD‐L1 expression and a non‐inflammatory tumor microenvironment [11]. Radiologically, these tumors often appear as nodules with ground‐glass opacity on CT, corresponding histologically to lepidic growth [12, 13]. Lung adenocarcinoma shows intratumoral heterogeneity, with mixed subtypes in ~80%–85% of cases [14, 15]. World Health Organization (WHO) classifies adenocarcinoma into Grade 1 (well‐differentiated, lepidic), Grade 2 (moderately differentiated, acinar, or papillary), and Grade 3 (poorly differentiated, with ≥ 20% high‐grade patterns). Identifying the predominant histological subtype is crucial, because it correlates with genetic and clinicopathological features [16, 17]. However, few studies have explored the relationship between PD‐L1‐high EGFRm NSCLC and its histological or radiological features.

This study aimed to identify EGFRm NSCLC patients who may benefit from ICI therapy using clinical data, including bronchoscopic biopsy and computed tomography (CT) imaging. To this end, we retrospectively analyzed advanced or recurrent EGFRm NSCLC cases to examine the relationship between PD‐L1 expression, histological subtype, and ICI efficacy and safety beyond second‐line treatment.

## 2 Materials and Methods

### 2.1 Patient Selection and Data Collection

This single‐center retrospective observational study was conducted at Hakodate Goryoukaku Hospital, Hokkaido, Japan. Consecutive treatment‐naïve patients with EGFRm NSCLC who received first‐line EGFR‐TKI (osimertinib or afatinib) between October 2018 and October 2023 were enrolled. Eligible patients had unresectable advanced or recurrent NSCLC. Exclusion criteria were prior EGFR‐TKI or ICI therapy, other malignancies, or missing data.

### 2.2 Patient Characteristics

Patient data included age, sex, performance status, smoking history, histopathology, stage, PD‐L1 expression, CT findings (within 4 weeks before treatment), treatment response, PFS, OS, post‐progression survival (PPS), time to treatment failure (TTF) of ICI, and primary tumor size. Tumor specimens obtained via cryobiopsy, needle biopsy, or forceps biopsy using bronchoscopy were evaluated by a board‐certified pathologist (K.A.) for subclassification.

Histological subtypes were defined according to the 2021 WHO classification: lepidic growth was defined as tumor cell proliferation along intact alveolar structures without architectural disruption; solid pattern as sheets of tumor cells lacking glandular differentiation; and papillary or acinar patterns according to WHO criteria. Radiologically, part‐solid GGNs were defined as lesions containing both ground‐glass and solid components, whereas solid nodules were defined as homogeneous soft‐tissue attenuation obscuring underlying lung markings.

Baseline tumor size was measured using RECIST v1.1, and PD‐L1 expression was assessed with the Dako PD‐L1 IHC 22C3 PharmDx test (SRL Inc., Tokyo, Japan) [18].

### 2.3 Definition and Assessment of Endpoints

Treatment response was assessed using RECIST v1.1. Severe pneumonitis was defined as ICI‐related toxicities (Grade ≥ 3 per CTCAE v5.0) or adverse effects leading to treatment discontinuation. PD‐L1 expression was determined using the Dako PD‐L1 IHC 22C3 PharmDx per manufacturer instructions [18].

PFS was measured from treatment start to lung cancer progression, death, or censoring. OS was measured from treatment start to death or censoring. PPS was calculated by subtracting PFS from OS in patients with progressive disease. TTF of ICI was defined as the time from ICI initiation to switching treatments. The last tumor assessment (for PFS) or clinic visit (for OS) before the end of follow‐up (January 2025) was used as the censoring date. Objective response rate was the percentage of confirmed complete or partial response per RECIST v1.1. Investigators measured all endpoints.

### 2.4 Statistical Analysis

Descriptive statistics summarized patient characteristics. The Mann–Whitney *U* test and Fisher's exact test assessed group differences. To adjust for confounding in first‐line analyses (e.g., Section 3.3), 1:1 propensity score matching (PSM) was performed using age, sex, smoking history, performance status, stage, mutation subtype, CNS metastasis, and first‐line EGFR‐TKI (caliper 0.2). PFS, OS, and TTF were compared using Kaplan–Meier curves and log‐rank tests; HRs were estimated with Cox models.

#### 2.4.1 Primary Analysis (Time‐Varying Cox)

For PPS from the first progression on first‐line EGFR‐TKI, we modeled ICI exposure as a time‐varying covariate in a Cox model with start–stop intervals. The time origin (*t*_0_) was the date of first progression on first‐line EGFR‐TKI. For each patient, follow‐up was split into (*t*_start, *t*_stop); ICI(*t*) = 0 before the first ICI dose and 1 thereafter (never‐treated remained 0). Same‐day ICI starts were represented by a single post‐ICI interval; intervals with *t*_stop ≤ *t*_start were excluded. This specification mitigates immortal‐time bias by aligning exposure with time at risk.

The prespecified model was:

$$\begin{array}{c} \text{Surv}\left(t\_\text{start},t\_\text{stop},\text{event}\right)\sim \mathrm{ICI}\left(t\right)\times \mathrm{PD}-L1\_\text{high}+\mathrm{age}\\ +\mathrm{sex}+\text{smoking}+\text{ECOG}\;\mathrm{PS}+\text{EGFR subtype}\\ +\text{brain metastasis}+\text{best response to 1L EGFR}\\ -\mathrm{TKI}\;\left(\mathrm{PR}/\mathrm{SD}/\mathrm{PD}\right)+\text{calendar year of progression} \end{array}$$

where PD‐L1_high was defined as tumor proportion score (TPS) ≥ 50% (vs. < 50%). All covariates were restricted to variables known by *t*_0_ (pre‐exposure). Ties were handled with the Efron method. Cluster‐robust (sandwich) standard errors at the patient level accounted for multiple intervals per subject. The ICI × PD‐L1 interaction was assessed by Wald tests and confirmed with a likelihood‐ratio test comparing models with and without the interaction. Stratum‐specific ICI effects (HRs within PD‐L1 strata) were obtained from linear contrasts of model coefficients.

#### 2.4.2 Model Diagnostics and Sensitivity Analyses

The proportional hazards (PH) assumption was evaluated using scaled Schoenfeld residuals (cox.zph). If non‐proportionality was indicated (e.g., for smoking history), we fit a stratified Cox model for that covariate as a sensitivity analysis. Robustness to early events before ICI exposure was examined using a 28‐day landmark analysis at *t*_0_. Missing data were handled by complete‐case analysis; zero‐length intervals were excluded a priori.

Analyses were performed using EZR (R version 4.4.1; The R Foundation, Vienna, Austria) and GraphPad Prism 10 (GraphPad Software, San Diego, CA, USA). Statistical significance was set at *p* < 0.05.

### 2.5 Ethical Considerations

This study was approved by the Institutional Review Board of Hakodate Goryoukaku Hospital (2024‐051, December 20, 2024) and adhered to the Declaration of Helsinki. An opt‐out consent process was used.

## 3 Results

### 3.1 Patient Inclusion and Characteristics

**FIGURE 1:**
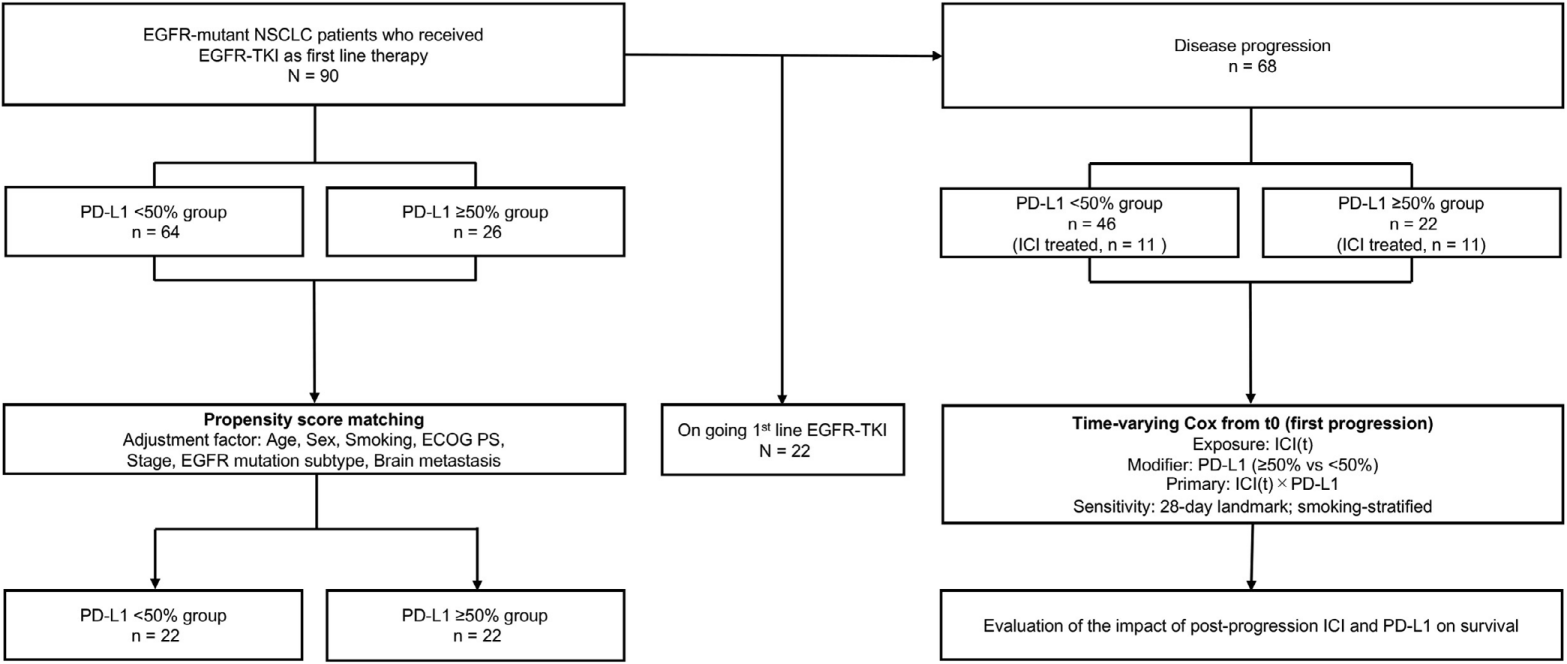
Study flow and analysis plan. Left branch (first‐line analyses): The full cohort of EGFR‐mutant NSCLC treated with first‐line EGFR‐TKI (*N* = 90) was stratified by PD‐L1 (TPS < 50% vs. ≥ 50%). For Section 3.3 outcomes, 1:1 propensity score matching was performed (age, sex, smoking, ECOG PS, stage, EGFR mutation subtype, Brain metastasis, first‐line EGFR‐TKI). Right branch (primary post‐progression analysis): Patients who progressed on first‐line EGFR‐TKI (*n* = 68) entered a time‐varying Cox model of post‐progression overall survival. Time origin *t*_0_ = date of first progression. Exposure ICI(*t*) = 0 before the first ICI dose and 1 thereafter (never‐treated remain 0). Effect modifier: PD‐L1 (≥ 50% vs. < 50%). The model adjusted only for pre‐*t*_0_ covariates (age, sex, smoking, ECOG PS, EGFR mutation subtype [Del19/L858R], brain metastasis, best response to 1L EGFR‐TKI [PR/SD/PD], calendar year of progression); Efron ties; patient‐cluster robust standard errors. Primary contrast: ICI(*t*) × PD‐L1 interaction. Sensitivity analyses: 28‐day landmark at *t*_0_; smoking‐stratified Cox. EGFR‐TKI, epidermal growth factor receptor–tyrosine kinase inhibitor; ICI, immune checkpoint inhibitor; PD‐L1, programmed death‐ligand 1; PD, progressive disease; PR, partial response; PS, performance status; SD, stable disease; TPS, tumor proportion score.

Ninety patients with advanced or recurrent NSCLC receiving first‐line EGFR‐TKI therapy between August 2018 and October 2023 were included, regardless of PD‐L1 expression. Patients were grouped by PD‐L1 expression: < 50% (*n* = 64) and ≥ 50% (*n* = 26) (Figure 1). Baseline characteristics are summarized in Table 1. The PD‐L1 ≥ 50% group had more advanced‐stage disease and a higher proportion with prior ICI use. The data cutoff was January 31, 2025, with a median follow‐up of 33.1 months (range: 1.3–73.5).

**TABLE 1:** Baseline characteristics of patients included in the study.

*n*		PD‐L1 < 50%	PD‐L1 ≥ 50%	*p*
		64	26	
Age (years)	(Range)	73 (51–93)	72 (43–83)	0.52
	< 75	33	15	0.65
	≥ 75	31	11	
Sex	Female	46	16	0.45
	Male	18	10	
PS	0	42	15	0.48
	≥ 1	22	11	
Stage	III/REC	17	5	0.59
	IV	47	21	
Smoking history	Never	38	12	0.35
	Former/current	26	14	
Mutation type	Del 19	37	11	0.24
	L858R	27	15	
PD‐L1	1%–49%	40	NA	
	< 1%	24	NA	
Metastatic site	Brain	18	10	0.45
	Liver	2	2	0.58
	Bone	16	11	0.13
	Pleura	17	6	0.79
First‐line treatment	Afatinib	24	10	1.0
	Osimertinib	40	16	

Abbreviations: Del19, exon 19 deletion; ICI, immune checkpoint inhibitor; L858R, exon 21 L858R; NA, not available; PD‐L1, programmed cell death ligand 1; REC, recurrence.

### 3.2 Correlation Between PD‐L1 Expression and Imaging/Histological Findings

**FIGURE 2:**
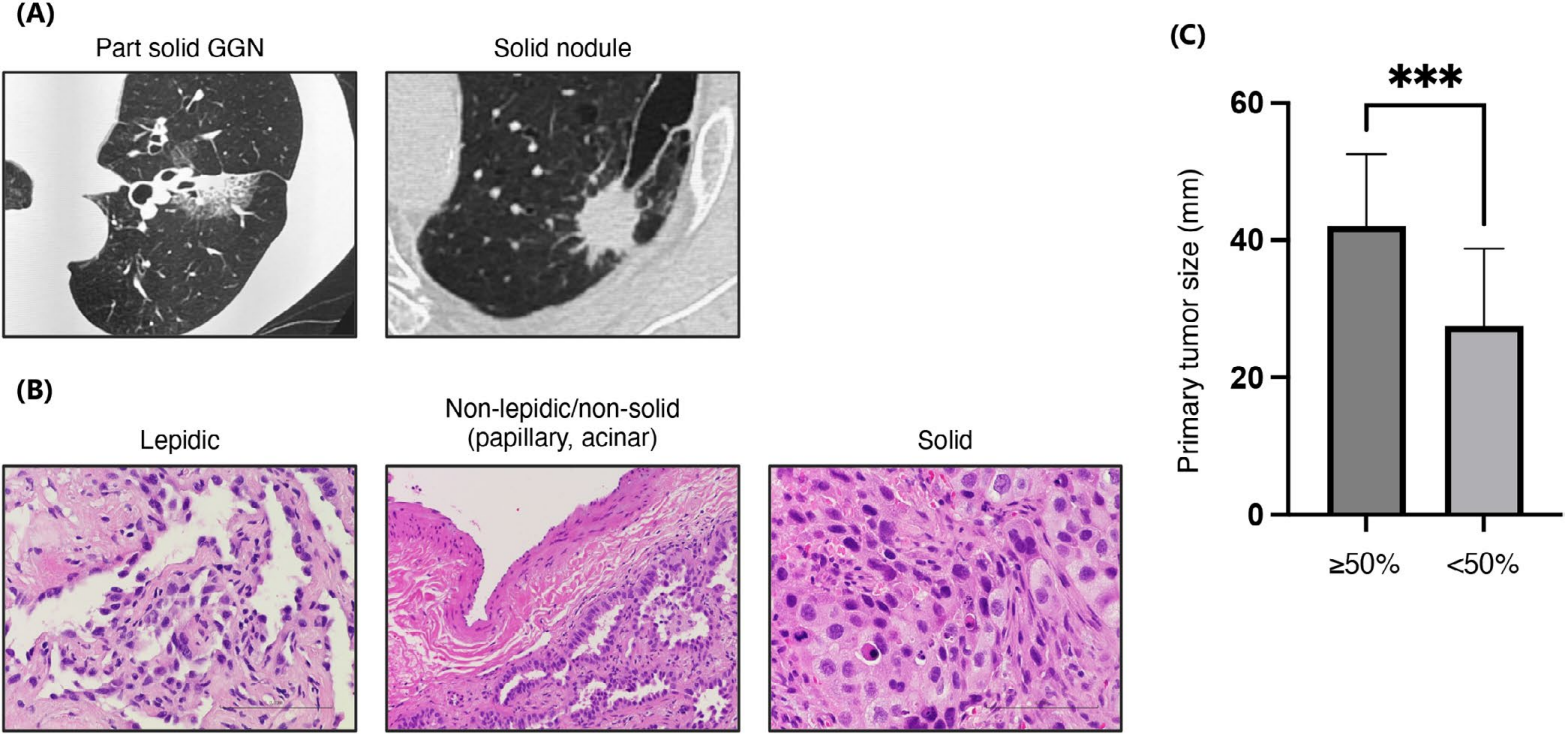
Imaging and pathological characteristics of EGFR‐mutant non‐small cell lung cancer. (A) Representative computed tomography images of part‐solid ground‐glass and solid nodules. (B) Representative histopathological images of specimens obtained from bronchoscopic biopsy, showing lepidic pattern, non‐lepidic or nonsolid, and solid patterns. (C) Classification of primary tumor maximum diameter based on PD‐L1 expression levels (PD‐L1 ≥ 50% vs. PD‐L1 < 50%). ***p < 0.001.

Associations between PD‐L1 expression, pathological, and radiological features are shown in Table 2; representative examples in Figure 2A,B. Tumors with a lepidic pattern were seen in 31% of the PD‐L1 < 50% group but none in the ≥ 50% group (*p* = 0.0005). Solid pattern tumors were more frequent in the PD‐L1 ≥ 50% group (62% vs. 8%, *p* < 0.0001). On CT, the PD‐L < 50% group had part‐solid ground‐glass nodules (GGN) and solid nodules in similar proportions (48% vs. 52%), while 96% of the PD‐L1 ≥ 50% group were solid nodules (*p* < 0.0001). Median primary tumor diameter was larger in the PD‐L1 ≥ 50% group (42.0 vs. 27.5 mm, *p* = 0.0007) (Figure 2C).

**TABLE 2:** PD‐L1 expression and histological/radiological findings.

	*n*	PD‐L1 < 50%	PD‐L1 ≥ 50%	*p*
		64	26	
Mutation	Del 19	37 (58)	11 (42)	0.24
	L858R	27 (42)	15 (58)	
Histological pattern	Lepidic	20 (31)	0 (0)	0.0005
	Non‐lepidic	44 (69)	26 (100)	
	Solid	5 (8)	16 (62)	< 0.0001
	Nonsolid	59 (92)	10 (38)	
CT pattern	Part solid GGN	31 (48)	1 (4)	< 0.0001
	Solid	33 (52)	25 (96)	

Abbreviations: CT, computed tomography; Del19, exon 19 deletion; GGN, ground‐glass nodule; ICI, immune checkpoint inhibitor; L858R, exon 21 L858R.

### 3.3 Response to First‐Line EGFR‐TKI Therapy and Correlation With PD‐L1 Expression, Histological Subtype, and Survival Outcomes

Responses to first‐line EGFR‐TKI by PD‐L1 expression are shown in Table 3. Disease control and response rates were lower in the PD‐L1 ≥ 50% group (81% vs. 97%, *p* = 0.02; 62% vs. 89%, *p* = 0.006). Seven patients showed primary resistance (progressive disease as best response), five from the PD‐L1 ≥ 50% group. Histological subtypes included three solid, three papillary, and one papillary with lepidic components (Table S1).

**TABLE 3:** PD‐L1 expression and the treatment efficacy of first‐line EGFR‐TKI therapy.

	*n*	PD‐L1 < 50%	PD‐L1 ≥ 50%	*p*
		64	26	
Best response	CR	2	0	0.011
	PR	55	16	
	SD	5	5	
	PD	2	5	
	Disease control rate	0.97	0.81	0.02
	Response rate	0.89	0.62	0.006

Abbreviations: CR, complete response; PD, progressive disease; PR, partial response; SD, stable disease.

**FIGURE 3:**
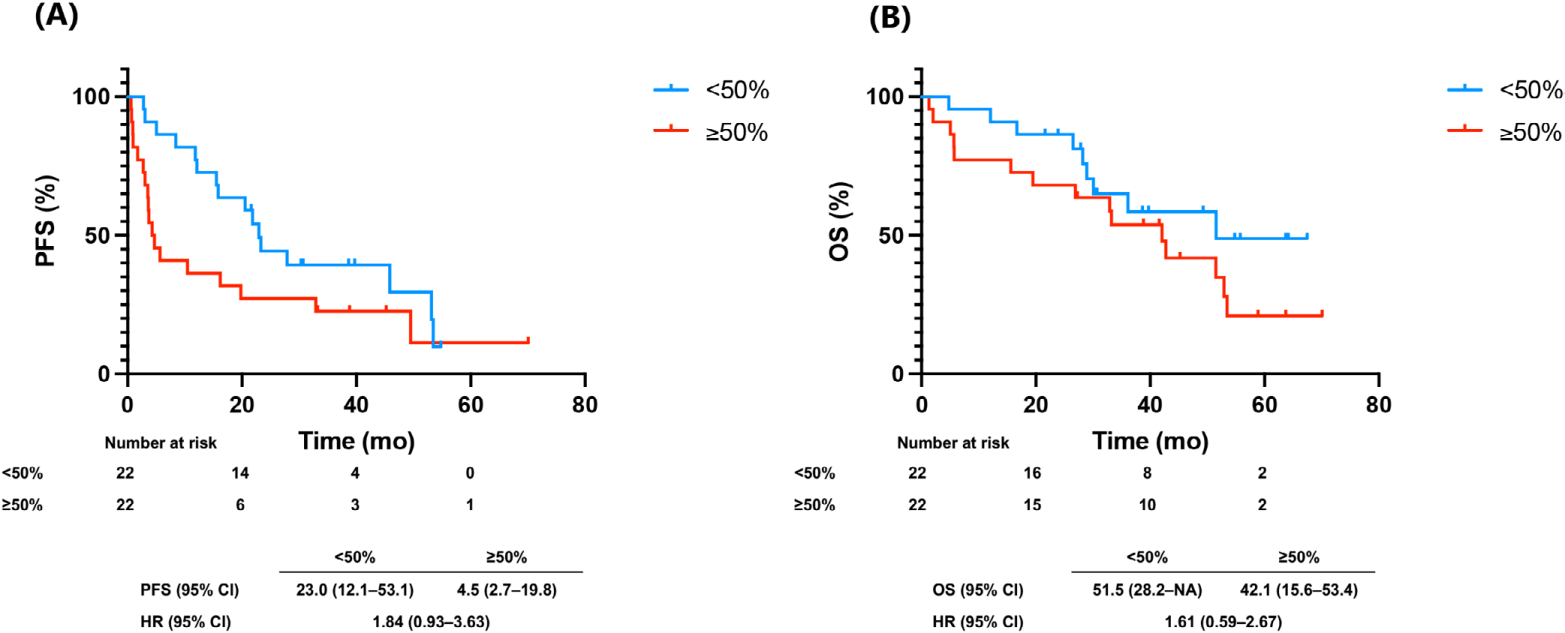
Progression‐free survival (PFS) and overall survival (OS) stratified by programmed death‐ligand 1 (PD‐L1) expression after propensity score matching. Adjustment factors included age, sex, smoking history, performance status, disease stage, epidermal growth factor receptor (EGFR) mutation subtype, central nervous system (CNS) metastasis, and first‐line EGFR‐tyrosine kinase inhibitor (EGFR‐TKI). (A) Progression‐free survival and (B) overall survival in the cohort of 44 patients after propensity score matching.

To control for baseline imbalances, we performed 1:1 PSM using age, sex, smoking, performance status, stage, mutation subtype, CNS metastasis, and first‐line EGFR‐TKI, yielding 22 matched pairs (Table S2). After PSM, median PFS was 23.0 months (95% CI: 12.1–53.1) in the PD‐L1 < 50% group versus 4.5 months (95% CI: 2.7–19.8) in the ≥ 50% group, showing a nonsignificant trend toward shorter PFS (HR: 1.84; 95% CI: 0.93–3.63, *p* = 0.074) (Figure 3A). Median OS was longer in the PD‐L1 < 50% group (51.5 months, 95% CI: 28.2–NA) versus the ≥ 50% group (42.1 months, 95% CI: 15.6–53.4), with a nonsignificant trend (HR 1.61; 95% CI: 0.59–2.67, *p* = 0.18) (Figure 3B). Unadjusted Kaplan–Meier curves are shown in Figure 1A,B.

### 3.4 PD‐L1 Expression, ICI Treatment History, and Overall Survival From First Progression (Post‐Progression OS/PPS)

Baseline characteristics at the first progression on first‐line EGFR‐TKI(*t*_0_) are summarized in Table S3 for all patients (All, *n* = 68) and those stratified by PD‐L1 (TPS < 50%: *n* = 46; ≥ 50%: *n* = 22). Distributions of age, ECOG performance status, smoking history, EGFR mutation subtype, and brain metastasis are shown with standardized mean differences (SMDs) to describe between‐group imbalance. Because post‐exposure variables (e.g., “ICI ever after *t*_0_” and “ICI at *t*_0_”) occur after the time origin, they are presented for description only and were not used for confounding adjustment.

**FIGURE 4:**
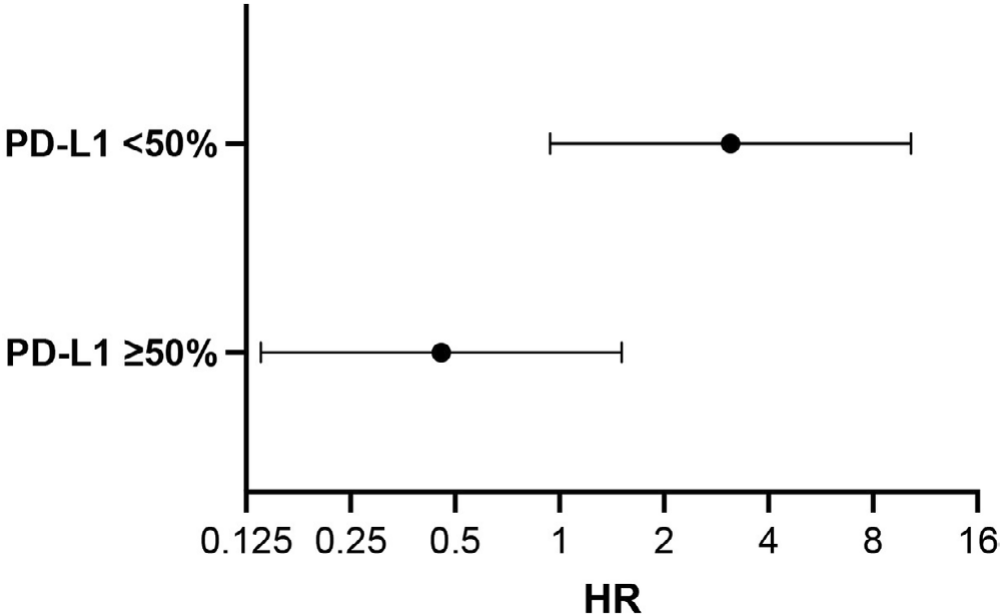
Effect of post‐progression ICI on survival by PD‐L1 stratum (time‐varying Cox). Forest plot of adjusted hazard ratios (HRs) for the association between post‐progression immune checkpoint inhibitor exposure and overall survival from the first progression on first‐line EGFR‐TKI(*t*_0_). ICI exposure was modeled as a time‐varying covariate ICI(*t*) (0 before the first ICI dose, 1 thereafter) in a start–stop Cox model. Points show HRs and horizontal bars 95% CIs on a log scale; the vertical line marks HR = 1 (no association). Estimates are adjusted for age, sex, smoking history, ECOG performance status, *EGFR* mutation subtype (Del19 vs. L858R), brain metastasis, best overall response to 1 L EGFR‐TKI (PR/SD/PD), and calendar year of progression, with patient‐cluster robust standard errors (Efron ties). PD‐L1 < 50%: HR: 3.11 (95% CI: 0.94–10.30), PD‐L1 ≥ 50%: HR: 0.457 (95% CI: 0.138–1.512). Interaction (ICI(*t*) × PD‐L1): Wald *p* = 0.015; likelihood‐ratio test *p* = 0.036. HR < 1 favors ICI; HR > 1 favors no ICI. CI, confidence interval; Del19, EGFR exon 19 deletion; ECOG PS, Eastern Cooperative Oncology Group performance status; EGFR‐TKI, epidermal growth factor receptor tyrosine kinase inhibitor; HR, hazard ratio; ICI, immune checkpoint inhibitor; L858R, EGFR exon 21 L858R; PD, progressive disease; PR, partial response; SD, stable disease; *t*_0_, date of first progression.

We analyzed OS from *t*_0_ using a time‐varying Cox model that treated post‐progression ICI as a time‐dependent covariate, thereby aligning exposure with risk time and mitigating immortal‐time bias. The ICI(*t*) × PD‐L1 interaction was statistically significant (Wald *p* = 0.015; likelihood‐ratio test *p* = 0.036), indicating effect modification by PD‐L1 (Figure 4, Table S4). Within PD‐L1 strata, the adjusted ICI effects were:

PD‐L1 < 50%: HR: 3.11 (95% CI: 0.94–10.30).

PD‐L1 ≥ 50%: HR: 0.457 (95% CI: 0.138–1.512).

The PHs assumption was not violated overall (global *p* = 0.515; Table S5). Although smoking showed a signal of non‐proportionality in diagnostics, a smoking‐stratified sensitivity model retained the significant interaction (LRT *χ* ^2^ = 6.06, df = 1, *p* = 0.0139). A 28‐day landmark analysis (0.92 months after *t*_0_) reproduced the interaction (Wald *p* = 0.0086), supporting robustness to early events prior to ICI exposure.

### 3.5 Efficacy of ICI Therapy and EGFR‐TKI Rechallenge According to PD‐L1 Expression

In an exploratory analysis, we evaluated all 22 ICI‐treated patients, regardless of PD‐L1 expression level. Baseline characteristics were similar between PD‐L1 < 50% and ≥ 50% groups (Table S6). These patients had more prior platinum‐based chemotherapy and multiple post‐EGFR‐TKI treatments. Disease control and response rates were 45% versus 64% (*p* = 0.67) and 9% versus 64% (*p* = 0.024), respectively, with a higher response in the PD‐L1 ≥ 50% group (Table S7). Median TTF was 1.4 months (95% CI: 0.7–2.8) in the PD‐L1 < 50% group vs. 3.4 months (95% CI: 0.9–13.3) in the ≥ 50% group (HRL 0.33; 95% CI: 0.12–0.92, *p* = 0.024) (Figure S2). Immune‐related adverse events included Grade 1–2 skin toxicity in eight patients (36%) and Grade 4 cytokine release syndrome in one.

We further analyzed the relationship between PFS with EGFR‐TKI and TTF with ICI in these 22 patients (Figure S3A). Among PD‐L ≥ 50% patients, 6 of 11 had longer TTF with ICI than PFS with EGFR‐TKI. In contrast, no PD‐L1 < 50% patient had TTF over 4 months or longer TTF with ICI than prior PFS. We also analyzed EGFR‐TKI rechallenge after ICI in 18 patients (Figure S3B). Among PD‐L1 < 50% patients, all but one had TTF < 3 months; among PD‐L1 ≥ 50% patients, 7 of 9 had TTF > 3 months, and 4 exceeded 10 months. Notably, four had longer TTF on EGFR‐TKI rechallenge than initial PFS.

## 4 Discussion

This study retrospectively analyzed the relationship between PD‐L1 expression, histopathological features, and therapeutic outcomes in EGFRm NSCLC to identify patients who may benefit from ICI therapy after EGFR‐TKI resistance. In the high PD‐L1 group (≥ 50%), (1) more tumors showed a solid histological pattern, (2) solid nodules predominated on CT, (3) response rates to EGFR‐TKIs were lower with shorter PFS, and (4) post‐progression ICI was differentially associated with PPS by PD‐L1 status, with a significant ICI(*t*) × PD‐L1 interaction in the time‐varying Cox model; exploratory analyses (ORR and TTF) were directionally consistent in the PD‐L1‐high subgroup. Exploratory analyses also suggested that EGFR‐TKI rechallenge after ICI therapy may be more effective in this group, highlighting the heterogeneity of EGFRm NSCLC and potential treatment implications.

EGFRm NSCLC is characterized by low PD‐L1 expression, high regulatory T cells (Treg) infiltration, and an immune‐cold tumor microenvironment [11]. Radiologically, these tumors often present as solid nodules with ground‐glass opacity [12], whereas histologically, they frequently exhibit a lepidic pattern [13]. Furthermore, previous studies have shown that PD‐L1 expression can correlate with increased tumor‐infiltrating lymphocytes [19, 20]. However, one of these studies also indicates that this correlation may be heterogeneous within tumors [20], suggesting that PD‐L1 alone does not fully capture the complexity of the immune microenvironment. This finding suggests that the immune landscape of EGFRm NSCLC is not uniformly cold. In this study, tumors with high PD‐L1 expression were more frequently associated with a solid histological pattern, whereas no tumors exhibited a lepidic pattern (*p* < 0.0001). These findings indicate that EGFRm NSCLC with high PD‐L1 expression may have more aggressive histopathological features. Furthermore, CT imaging showed that 96% of the tumors in the PD‐L1‐high group were solid nodules with minimal ground‐glass opacity (*p* < 0.0001). These results suggest that PD‐L1 expression in EGFRm NSCLC is associated with tumor progression patterns and histological characteristics. Although this study focused on PD‐L1 expression, the EGFRm NSCLC responsiveness to ICI may also be associated with other biomarkers, such as tumor mutational burden, *STK11*/*KEAP1* mutations, and tumor‐infiltrating lymphocyte density. Recent studies have reported that EGFRm NSCLC harboring *STK11*/*KEAP1* mutations exhibits an immunosuppressive tumor microenvironment, leading to poor responsiveness to ICIs and reduced efficacy of osimertinib [21]. Future studies should incorporate comprehensive biomarker analyses, including next‐generation sequencing for genetic profiling, tumor mutational burden assessment, and tumor‐infiltrating lymphocyte analysis, to more accurately identify patients most likely to benefit from ICI therapy.

In the primary time‐varying Cox analysis that aligned ICI exposure with time at risk, the ICI(*t*) × PD‐L1 interaction was significant, with stratum‐specific adjusted effects suggesting a detrimental association of ICI in PD‐L1 < 50% (HR: 3.11, 95% CI: 0.94–10.30) but a favorable association in PD‐L1 ≥ 50% (HR: 0.457, 95% CI: 0.138–1.512). While CIs within strata were wide, the between‐strata difference supports PD‐L1 as an effect modifier. Consistent with this pattern, the PD‐L1‐high group showed higher ICI response rates and longer TTF. Because PPS metrics such as PPS are vulnerable to selection and immortal‐time biases, we refrain from causal inference based on PPS. Recent case reports and subgroup analyses have suggested potential benefit in PD‐L1‐high EGFRm NSCLC [7, 22, 23, 24, 25], whereas KEYNOTE‐789 found no survival advantage for adding ICI to chemotherapy in the overall population or in TPS ≥ 50% [26]. Given these conflicting results across studies, the correlation between PD‐L1 levels and ICI response remains inconclusive and warrants further investigation. These discrepancies may be attributed to differences in patient populations, specific ICI agents used, the amount of VEGF inhibitor added, or variations in PD‐L1 assessment methodologies. A prospective study using a standardized PD‐L1 evaluation protocol targeting PD‐L1 ≥ 50% is needed to clarify this relationship.

Among the 22 patients who received ICI therapy in this study, many had undergone multiple lines of treatment, including platinum‐based combination chemotherapy. Notably, some patients exhibited favorable responses to EGFR‐TKI retreatment following ICI therapy. Previous studies have reported that PD‐L1 expression levels increase after EGFR‐TKI resistance, potentially enhancing the response to subsequent immunotherapy [27]. Additionally, EGFR‐TKIs have been shown to alter the tumor microenvironment by reducing Treg suppressive activity and enhancing cytotoxic T‐cell activation [28]. In some patients, combination therapy with ICIs and EGFR‐TKIs resulted in prolonged response durations [29]. These findings suggest that the tumor microenvironment in EGFRm NSCLC may evolve during treatment, and sequential administration of ICIs followed by EGFR‐TKI retreatment may synergistically affect tumor immunity.

A possible immunologic explanation of our findings is that the mechanisms underlying response differ between initial EGFR‐TKI therapy and EGFR‐TKI rechallenge. Initial EGFR‐TKI therapy primarily targets oncogene‐addicted tumor cell signaling and is often more effective in PD‐L1–low tumors. In contrast, EGFR‐TKI retreatment after ICI exposure may exert antitumor activity not only through direct inhibition of EGFR signaling but also by acting on an ICI‐modulated tumor microenvironment—such as reduced Treg suppressive activity, increased CD8+ T‐cell infiltration, or reprogramming of adaptive immune resistance pathways. This dual mechanism may help explain why patients with high PD‐L1 expression experienced more favorable outcomes in response to EGFR‐TKI retreatment in our cohort.

In this study, the prolonged PPS observed in patients with PD‐L1 ≥ 50% who received ICI therapy may be attributed, in part, to the favorable response to EGFR‐TKI retreatment. Based on these findings, future studies should focus on dynamic analyses of the tumor immune microenvironment, including variations in PD‐L1 expression and changes in tumor‐infiltrating lymphocytes, to further elucidate the mechanisms underlying the effectiveness of sequential ICI and EGFR‐TKI therapies. These observations are hypothesis‐generating and may reflect selection and indication processes inherent to retrospective sequencing analyses.

This study provides important insights into the relationship between PD‐L1 expression and ICI efficacy; however, some limitations should be considered. Because this was a retrospective single‐center study, generalizability to other populations and clinical settings may be limited. The number of ICI‐treated patients was small, resulting in wide confidence intervals for the stratum‐specific HRs and limited precision of the estimates. Moreover, because ICI allocation was non‐randomized, substantial confounding by indication cannot be excluded. Although we restricted covariates to those known at *t*_0_ to preserve causal ordering and mitigate collider bias using a time‐varying Cox model, unmeasured factors such as comorbidity burden, disease aggressiveness, and physician treatment preference may have influenced treatment selection and outcomes. We used complete‐case analysis, which may introduce bias if missingness was not at random. Finally, PD‐L1 assessment was performed on limited biopsy specimens, which may not fully capture intratumoral spatial heterogeneity.

## 5 Conclusions

In EGFRm NSCLC, PD‐L1‐high tumors exhibited solid histology and solid CT morphology more frequently. Using a time‐varying Cox analysis from the first progression, we found that PD‐L1 modified the association between post‐progression ICI and survival: the adjusted ICI effect tended to be favorable in PD‐L ≥ 50% and unfavorable in PD‐L1 < 50%, with a significant interaction and supportive sensitivity analyses. These data support PD‐L1‐informed selection of post‐TKI therapy and motivate prospective studies to confirm effect modification and define optimal sequencing strategies.

## Author Contributions

Toshiyuki Sumi made substantial contributions to the conception of the work, made significant contributions to the design of the work and the interpretation of data, and drafted the original manuscript. Kotomi Arioka made significant contributions to the data analysis and interpretation. Hirofumi Chiba and other authors substantially contributed to the revision of the manuscript drafts. All authors have approved the submitted version of the manuscript and agreed to be accountable for any part of the work.

## Funding

The authors have nothing to report.

## Conflicts of Interest

Dr. Toshiyuki Sumi received lecture fees from Chugai Pharmaceutical Co., Nippon Boehringer Ingelheim Co., Ono Pharmaceutical, and AstraZeneca. Dr. Hirofumi Chiba received a lecture fee from Nippon Boehringer Ingelheim Co. The other authors declare no conflicts of interest.

## Supporting information


**Figure S1:** Unadjusted Kaplan–Meier survival curves for progression‐free survival (PFS) and overall survival (OS) stratified by programmed death‐ligand 1 expression in the overall cohort before propensity score matching. (A) PFS and (B) OS in the overall cohort of 90 patients. HR, hazard ratio; mo, month; OS, overall survival; PFS, progression free survival.
**Figure S2:** Time to treatment failure stratified by programmed death‐ligand 1 expression in patients treated with immune checkpoint inhibitors (ICIs).
**Figure S3:** Swimmer plots illustrating treatment outcomes in patients receiving immune checkpoint inhibitors (ICIs). (A) Comparison between time to treatment failure (TTF) with ICI therapy and progression‐free survival (PFS) with first‐line epidermal growth factor receptor‐tyrosine kinase inhibitor (EGFR‐TKI) therapy in 22 patients. (B) Comparison between TTF with EGFR‐TKI rechallenge after ICI therapy and PFS with first‐line EGFR‐TKI therapy in 18 patients stratified by programmed death‐ligand 1 (PD‐L1) expression. ICI, immune‐checkpoint inhibitor; mo, month; OS, overall survival; PD‐L1, programmed death‐ligand 1; PFS, progression free survival; Pt, patient; TKI, tyrosine kinase inhibitor; TTF, time to treatment failure.

**Table S1:** Background characteristics of seven patients resistant to the first‐line of epidermal growth factor receptor‐tyrosine kinase inhibitors therapy.
**Table S2:** Patient characteristics stratified by PD‐L1 expression status after propensity score matching.
**Table S3:** Baseline characteristics at first progression on first‐line EGFR‐TKI(*t*_0_): All patients and stratified by PD‐L1 tumor proportion score (TPS < 50% vs ≥ 50%).
**Table S4:** Adjusted hazard ratios from the primary time‐varying Cox model for post‐progression OS/PPS (*t*_0_ = first progression) including the ICI(*t*) × PD‐L1 interaction.
**Table S5:** Proportional hazards (PH) diagnostics by scaled Schoenfeld residuals for the primary time‐varying Cox model of post‐progression OS/PPS (*t*_0_ = first progression).
**Table S6:** Patient characteristics stratified by PD‐L1 expression in 22 patients who receive ICI therapy in second‐line or later treatments.
**Table S7:** PD‐L1 expression and the treatment efficacy of ICI therapy in second‐line or later treatments.

## Data Availability

The data that support the findings of this study are available on request from the corresponding author. The data are not publicly available due to privacy or ethical restrictions.
