# Supplementary material for: PD‐L1 Expression and Histopathological Features in EGFR‐Mutated Non‐Small Cell Lung Cancer: Implications for Immune Checkpoint Inhibitors After EGFR‐Tyrosine Kinase Inhibitors Resistance
Source: Thorac Cancer. 2026 Feb 9;17(3):e70252. doi: 10.1111/1759-7714.70252 (PMC12885618; doi:10.1111/1759-7714.70252)
Supplement: Supplementary file 1 — Figure S1: Unadjusted Kaplan–Meier survival curves for progression‐free survival (PFS) and overall survival (OS) stratified by programmed death‐ligand 1 expression in the overall cohort before propensity score matching. (A) PFS and (B) OS in the overall cohort of 90 patients. HR, hazard ratio; mo, month; OS, overall survival; PFS, progression free survival. Figure S2: Time to treatment failure stratified by programmed death‐ligand 1 expression in patients treated with immune checkpoint inhibitors (ICIs). Figure S3: Swimmer plots illustrating treatment outcomes in patients receiving immune checkpoint inhibitors (ICIs). (A) Comparison between time to treatment failure (TTF) with ICI therapy and progression‐free survival (PFS) with first‐line epidermal growth factor receptor‐tyrosine kinase inhibitor (EGFR‐TKI) therapy in 22 patients. (B) Comparison between TTF with EGFR‐TKI rechallenge after ICI therapy and PFS with first‐line EGFR‐TKI therapy in 18 patients stratified by programmed death‐ligand 1 (PD‐L1) expression. ICI, immune‐checkpoint inhibitor; mo, month; OS, overall survival; PD‐L1, programmed death‐ligand 1; PFS, progression free survival; Pt, patient; TKI, tyrosine kinase inhibitor; TTF, time to treatment failure. [file TCA-17-e70252-s002.docx]

**Supplementary Figures**

**Supplementary Figure 1.** Unadjusted Kaplan–Meier survival curves for progression-free survival (PFS) and overall survival (OS) stratified by programmed death-ligand 1 expression in the overall cohort before propensity score matching. (A) PFS and (B) OS in the overall cohort of 90 patients.
Abbreviations: PFS, progression free survival; OS, overall survival; mo, month; HR, hazard ratio.

**Supplementary Figure 2.** Time to treatment failure stratified by programmed death-ligand 1 expression in patients treated with immune checkpoint inhibitors (ICIs).

Abbreviations: TTF, time to treatment failure; mo, month; HR, hazard ratio.

**Supplementary Figure 3.** Swimmer plots illustrating treatment outcomes in patients receiving immune checkpoint inhibitors (ICIs). (A) Comparison between time to treatment failure (TTF) with ICI therapy and progression-free survival (PFS) with first-line epidermal growth factor receptor-tyrosine kinase inhibitor (EGFR-TKI) therapy in 22 patients. (B) Comparison between TTF with EGFR-TKI re-challenge after ICI therapy and PFS with first-line EGFR-TKI therapy in 18 patients stratified by programmed death-ligand 1 (PD-L1) expression.
Abbreviations: Pt, patient; PFS, progression free survival; OS, overall survival; mo, month; PD-L1, programmed death-ligand 1; TKI, tyrosine kinase inhibitor; TTF, time to treatment failure; ICI, immune-checkpoint inhibitor.
