# Supplementary material for: PD‐L1 Expression and Histopathological Features in EGFR‐Mutated Non‐Small Cell Lung Cancer: Implications for Immune Checkpoint Inhibitors After EGFR‐Tyrosine Kinase Inhibitors Resistance
Source: Thorac Cancer. 2026 Feb 9;17(3):e70252. doi: 10.1111/1759-7714.70252 (PMC12885618; doi:10.1111/1759-7714.70252)
Supplement: Supplementary file 2 — Table S1: Background characteristics of seven patients resistant to the first‐line of epidermal growth factor receptor‐tyrosine kinase inhibitors therapy. Table S2: Patient characteristics stratified by PD‐L1 expression status after propensity score matching. Table S3: Baseline characteristics at first progression on first‐line EGFR‐TKI(t 0): All patients and stratified by PD‐L1 tumor proportion score (TPS < 50% vs ≥ 50%). Table S4: Adjusted hazard ratios from the primary time‐varying Cox model for post‐progression OS/PPS (t 0 = first progression) including the ICI(t) × PD‐L1 interaction. Table S5: Proportional hazards (PH) diagnostics by scaled Schoenfeld residuals for the primary time‐varying Cox model of post‐progression OS/PPS (t 0 = first progression). Table S6: Patient characteristics stratified by PD‐L1 expression in 22 patients who receive ICI therapy in second‐line or later treatments. Table S7: PD‐L1 expression and the treatment efficacy of ICI therapy in second‐line or later treatments. [file TCA-17-e70252-s001.docx]

**Supplementary Tables**

**Supplementary Table 1. Background characteristics of seven patients resistant to the first-line of epidermal growth factor receptor-tyrosine kinase inhibitors therapy**

| **Patient(s)** | **Sex** | **Age**  **(years)** | **Stage** | **Type** | **PD-L1** | **1st line Tx** | **PFS (m)** | **CT pattern** | **Histology** |
| --- | --- | --- | --- | --- | --- | --- | --- | --- | --- |
| 1 | Female | 62 | REC | L858R | <1％ | AFA | 0.5 | Part solid GGN | Lepidic+papillary |
| 2 | Female | 77 | IV | L858R | ≥50% | OSI | 0.6 | Solid | Papillary |
| 3 | Female | 70 | IV | Del19 | 1-49％ | OSI | 0.9 | Solid | Solid |
| 4 | Female | 83 | IV | L858R | ≥50% | OSI | 1 | Part solid GGN | Papillary |
| 5 | Female | 69 | IV | L858R | ≥50% | OSI | 1 | Solid | Solid |
| 6 | Male | 73 | IV | Del19 | ≥50% | OSI | 1.8 | Solid | Solid |
| 7 | Female | 77 | IV | L858R | ≥50% | OSI | 3 | Part solid GGN | Papillary |

Abbreviations: PD-L1, programmed cell death ligand 1; Tx, treatment; PFS, progression-free survival; m, month; CT, computed tomography; REC, recurrence; Del19, exon 19 deletion; L858R, exon 21 L858R; AFA, afatinib; OSI, osimertinib; GGN, ground-glass nodule.

**Supplementary Table 2. Patient characteristics stratified by PD-L1 expression status after propensity score matching**

|  |  | **PD-L1 <50%** |  | **PD-L1 ≥50%** |  |  |
| --- | --- | --- | --- | --- | --- | --- |
| **n** |  | **22** |  | **22** |  | **p value** |
| Age (years) | <75 | 14 |  | 13 |  | 0.77 |
|  | ≥75 | 8 |  | 9 |  |  |
| Sex | Female | 15 |  | 13 |  | 0.76 |
|  | Male | 7 |  | 9 |  |  |
| Stage | III/REC | 4 |  | 5 |  | 1 |
|  | IV | 18 |  | 17 |  |  |
| Smoking history | never | 13 |  | 11 |  | 0.76 |
|  | former/current | 9 |  | 11 |  |  |
| PS | 0 | 15 |  | 14 |  | 1 |
|  | ≥1 | 7 |  | 8 |  |  |
| Mutation subtype | Del 19 | 9 |  | 9 |  | 1 |
|  | L858R | 13 |  | 13 |  |  |
| PD-L1 | 1–49% | 15 |  | NA |  |  |
|  | <1% | 7 |  | NA |  |  |
| Metastatic site | Brain | 6 |  | 7 |  | 1 |
|  | Liver | 1 |  | 2 |  | 1 |
|  | Bone | 4 |  | 9 |  | 0.19 |
|  | Pleura | 9 |  | 5 |  | 0.33 |
| Furst-line treatment | Afatinib | 9 |  | 9 |  | 1 |
|  | Osimertinib | 13 |  | 13 |  |  |

Adjustment factor: Age, Sex, Smoking, PS, Stage, mutation, CNS meta, first-line epidermal growth factor receptor-tyrosine kinase inhibitors

Abbreviations: PD-L1, programmed cell death ligand 1; REC, recurrence; Del19, exon 19 deletion; L858R, exon 21 L858R.

**Supplementary Table 3. Baseline characteristics at first progression on first-line EGFR-TKI (t0): All patients and stratified by PD-L1 tumor proportion score (TPS <50% vs ≥50%)**

| **Variable** | **All**  **(n=68)** | **PD-L1 <50% (n=46)** | **PD-L1 ≥50% (n=22)** | **SMD** |
| --- | --- | --- | --- | --- |
| **n** | **68** | **46** | **22** |  |
| Age, years (mean [SD]) | 73.16 (8.41) | 73.65 (8.11) | 72.14 (9.12) | 0.17 |
| Sex — Female, n (%) | 44 (64.7%) | 32 (69.6%) | 12 (54.5%) | 0.31 |
| Sex — Male, n (%) | 24 (35.3%) | 14 (30.4%) | 10 (45.5%) | 0.31 |
| ECOG PS — 0, n (%) | 43 (63.2%) | 29 (63.0%) | 14 (63.6%) | 0.01 |
| ECOG PS — ≥1, n (%) | 25 (36.8%) | 17 (37.0%) | 8 (36.4%) | 0.01 |
| Smoking history — No, n (%) | 38 (55.9%) | 29 (63.0%) | 9 (40.9%) | 0.45 |
| Smoking history — Yes, n (%) | 30 (44.1%) | 17 (37.0%) | 13 (59.1%) | 0.45 |
| EGFR mutation subtype — Del19, n (%) | 35 (51.5%) | 26 (56.5%) | 9 (40.9%) | 0.32 |
| EGFR mutation subtype — L858R, n (%) | 33 (48.5%) | 20 (43.5%) | 13 (59.1%) | 0.32 |
| Brain metastasis — No, n (%) | 47 (69.1%) | 35 (76.1%) | 12 (54.5%) | 0.46 |
| Brain metastasis — Yes, n (%) | 21 (30.9%) | 11 (23.9%) | 10 (45.5%) | 0.46 |
| Best response to 1L EGFR-TKI — PD, n (%) | 7 (10.3%) | 2 (4.3%) | 5 (22.7%) | 0.56 |
| Best response to 1L EGFR-TKI — PR, n (%) | 52 (76.5%) | 40 (87.0%) | 12 (54.5%) | 0.76 |
| Best response to 1L EGFR-TKI — SD, n (%) | 9 (13.2%) | 4 (8.7%) | 5 (22.7%) | 0.39 |
| Calendar year of progression (mean [SD]) | 2021.78 (1.63) | 2022.11 (1.69) | 2021.09 (1.27) | 0.68 |
| ICI ever after t0 — No, n (%) | 46 (67.6%) | 35 (76.1%) | 11 (50.0%) |  |
| ICI ever after t0 — Yes, n (%) | 22 (32.4%) | 11 (23.9%) | 11 (50.0%) |  |
| ICI at t0 — No, n (%) | 65 (95.6%) | 45 (97.8%) | 20 (90.9%) |  |
| ICI at t0 — Yes, n (%) | 3 (4.4%) | 1 (2.2%) | 2 (9.1%) |  |

Abbreviations: PD-L1, programmed death-ligand 1; TPS, tumor proportion score; EGFR-TKI, epidermal growth factor receptor tyrosine kinase inhibitor; ECOG PS, Eastern Cooperative Oncology Group performance status; Del19, EGFR exon 19 deletion; L858R, EGFR exon 21 L858R; PR, partial response; SD, stable disease; PD, progressive disease; ICI, immune checkpoint inhibitor; t0, date of first progression on first-line EGFR-TKI (time origin); SMD, standardized mean difference; 1L, first-line; TPS, tumor proportion score.

Notes: SMDs are shown to describe between-group imbalance (|SMD|≈0.10 small, ≈0.20 moderate).

“ICI ever after t0” and “ICI at t0” occur after the time origin and are presented for description only (not used for confounding adjustment).

**Supplementary Table 4. Adjusted hazard ratios from the primary time-varying Cox model for post-progression OS/PPS (t0 = first progression) including the ICI(t) × PD-L1 interaction**

| **Covariate** | **HR** | **95% CI (L)** | **95% CI (U)** | **p value** |
| --- | --- | --- | --- | --- |
| ICI(t) | 3.11 | 0.94 | 10.30 | 0.06 |
| PD-L1 ≥50% (vs. <50%) | 2.63 | 1.30 | 5.34 | 0.01 |
| Age (per year) | 1.07 | 1.01 | 1.13 | 0.03 |
| Smoking (Yes vs No) | 1.34 | 0.43 | 4.21 | 0.61 |
| Sex (Male vs Female) | 0.24 | 0.09 | 0.66 | 0.01 |
| ECOG PS ≥1 (vs. 0) | 2.10 | 1.15 | 3.82 | 0.02 |
| EGFR L858R (vs. Del19) | 2.24 | 0.78 | 6.45 | 0.13 |
| Brain metastasis (Yes) | 1.98 | 0.92 | 4.23 | 0.08 |
| BOR: PR vs. PD | 1.18 | 0.39 | 3.60 | 0.77 |
| BOR: SD vs. PD | 0.84 | 0.30 | 2.38 | 0.74 |
| Calendar year of PD | 1.04 | 0.82 | 1.31 | 0.77 |
| Interaction ICI×PD-L1 | 0.15 | 0.03 | 0.69 | 0.02 |

Abbreviations:

PD-L1, programmed death-ligand 1; TPS, tumor proportion score; EGFR-TKI, epidermal growth factor receptor tyrosine kinase inhibitor; ECOG PS, Eastern Cooperative Oncology Group performance status; EGFR Del19, EGFR exon 19 deletion; EGFR L858R, EGFR exon 21 L858R; BOR, best overall response to 1L EGFR-TKI; ICI, immune checkpoint inhibitor; ICI(t), time-varying ICI exposure; OS, overall survival; PPS, post-progression survival; HR, hazard ratio; CI, confidence interval; t0, date of first progression on 1L EGFR-TKI (time origin); 1L, first-line.

Notes:

Outcome is overall survival from t0 (post-progression OS/PPS).

ICI(t) is coded 0 before the first ICI dose and 1 after; never-treated remain 0.

The ICI(t) main effect represents the ICI hazard ratio within PD-L1 <50% (reference stratum).

The Interaction (ICI×PD-L1) modifies the ICI effect in PD-L1 ≥50%; the stratum-specific HR for PD-L1 ≥50% equals exp[ log(HR_ICI) + log(HR_interaction) ] (reported in the text/figure as HR 0.457, 95% CI 0.138–1.512).

Covariates were restricted to variables known at t0 to preserve causal ordering.

Cox model used Efron method for ties and patient-cluster robust (sandwich) SEs.

BOR categories are relative to PD: “PR vs PD” and “SD vs PD.”

“Calendar year of PD” denotes the year of first progression on 1L EGFR-TKI.**Supplementary Table 5. Proportional hazards (PH) diagnostics by scaled Schoenfeld residuals for the primary time-varying Cox model of post-progression OS/PPS (t0 = first progression)**

| **Covariate** | **p-value** |
| --- | --- |
| ICI(t) | 0.322 |
| PD-L1 ≥50% | 0.297 |
| Age | 0.203 |
| Smoking history | 0.025 |
| Sex | 0.084 |
| ECOG PS | 0.132 |
| EGFR L858R | 0.154 |
| Brain metastasis | 0.367 |
| BOR (overall) | 0.452 |
| Calendar year of PD | 0.979 |
| Interaction ICI×PD-L1 | 0.216 |
| GLOBAL | 0.515 |

Abbreviations: PH, proportional hazards; PD-L1, programmed death-ligand 1; ECOG PS, Eastern Cooperative Oncology Group performance status; EGFR, epidermal growth factor receptor; Del19, EGFR exon 19 deletion; L858R, EGFR exon 21 L858R; BOR, best overall response to 1L EGFR-TKI; ICI, immune checkpoint inhibitor; ICI(t), time-varying ICI exposure; PD, progressive disease; PR, partial response; SD, stable disease; t0, date of first progression on 1L EGFR-TKI.

Notes:

p-values test the PH assumption for each covariate (and GLOBAL for the model). p<0.05 suggests potential PH violation.

“BOR (overall)” is a joint test across BOR categories.

In these data, Smoking history showed evidence of non-proportionality (p=0.025), whereas the GLOBAL test does not indicate overall violation (p=0.515); a smoking-stratified sensitivity model is therefore performed.

**Supplementary Table 6. Patient characteristics stratified by PD-L1 expression in 22 patients who receive ICI therapy in second-line or later treatments**

|  |  | **PD-L1 <50%** | **PD-L1 ≥50%** |  |
| --- | --- | --- | --- | --- |
|  | **n** | **11** | **11** | **p value** |
| Age (years) | <75 | 8 | 9 | 1 |
|  | ≥75 | 3 | 2 |  |
| Sex | Female | 6 | 1 | 0.063 |
|  | Male | 5 | 10 |  |
| Stage | III/REC | 1 | 3 | 0.59 |
|  | IV | 10 | 8 |  |
| Mutation type | Del 19 | 7 | 5 | 0.67 |
|  | L858R | 4 | 6 |  |
| Pretreatment | Afatinib | 6 | 8 | 0.66 |
|  | Osimertinib | 5 | 3 |  |
| Pretreatment BOR | CR/PR | 9 | 9 | 1 |
|  | SD | 1 | 1 |  |
|  | PD | 1 | 1 |  |
| Platinum doublet treatment |  | 9 | 11 | 0.77 |
| ICI treatment line |  | 3 (2–5) | 3 (2–5) | 0.65 |
| Atezo, BEV, CBDCA+PTX |  | 1 | 3 | 0.33 |
| Atezo |  | 7 | 8 |  |
| NIVO |  | 1 | 0 |  |
| Pemb |  | 2 | 0 |  |
| Treatment line post EGFR-TKI progression | | 4 (3–6) | 4 (2–6) | 0.48 |

Abbreviations: REC, recurrence; Del19, exon 19 deletion; L858R, exon 21 L858R; BOR, best of response; CR, complete response; PD-L1, programmed death-ligand 1; PR, partial response; SD, stable disease; PD, progressive disease; ICI, immune checkpoint inhibitor; Atezo, atezolizumab; BEV, bevacizumab; CBDCA, carboplatin; PTX, paclitaxel; NIVO, nivolumab; Pemb, pembrolizumab; EGFR-TKI, epidermal growth factor receptor tyrosine kinase inhibitor.

**Supplementary Table 7. PD-L1 expression and the treatment efficacy of ICI therapy in second-line or later treatments**

|  |  | **PD-L1 <50%** | **PD-L1 ≥50%** |  |
| --- | --- | --- | --- | --- |
|  | **n** | **11** | **11** | **p value** |
| Best response | CR | 0 | 1 | 0.016 |
|  | PR | 1 | 6 |  |
|  | SD | 4 | 0 |  |
|  | PD | 6 | 4 |  |
|  | Disease control rate | 0.45 | 0.64 | 0.67 |
|  | Response rate | 0.09 | 0.64 | 0.024 |

Abbreviations: ICI, immune checkpoint inhibitors; CR, complete response; PR, partial response; PD-L1, programmed death-ligand 1; SD, stable disease; PD, progressive disease.
